# Effects of cash transfers on Children’s health and social protection in Sub-Saharan Africa: differences in outcomes based on orphan status and household assets

**DOI:** 10.1186/s12889-015-1857-4

**Published:** 2015-05-28

**Authors:** Thomas M. Crea, Andrew D. Reynolds, Aakanksha Sinha, Jeffrey W. Eaton, Laura A. Robertson, Phyllis Mushati, Lovemore Dumba, Gideon Mavise, J. C. Makoni, Christina M. Schumacher, Constance A. Nyamukapa, Simon Gregson

**Affiliations:** School of Social Work, Boston College, 140 Commonwealth Ave., Chestnut Hill, Massachusetts, 01746 USA; School of Public Health, Imperial College London, London, W2 1PG UK; Biomedical Research and Training Institute, Harare, Zimbabwe; Department of Social Services, London, Zimbabwe; Catholic Relief Services Zimbabwe, Kenya Plaza, 95 Park Lane, London, Zimbabwe; Diocese of Mutare Community Care Program (DOMCCP), St. Joseph’s Mission, P.O. Box 43, Mutare, Zimbabwe

**Keywords:** Cash transfers, Social protection, Orphaned and vulnerable children, Sub-Saharan Africa, HIV/AIDS

## Abstract

**Background:**

Unconditional and conditional cash transfer programmes (UCT and CCT) show potential to improve the well-being of orphans and other children made vulnerable by HIV/AIDS (OVC). We address the gap in current understanding about the extent to which household-based cash transfers differentially impact individual children’s outcomes, according to risk or protective factors such as orphan status and household assets.

**Methods:**

Data were obtained from a cluster-randomised controlled trial in eastern Zimbabwe, with random assignment to three study arms – UCT, CCT or control. The sample included 5,331 children ages 6-17 from 1,697 households. Generalized linear mixed models were specified to predict OVC health vulnerability (child chronic illness and disability) and social protection (birth registration and 90% school attendance). Models included child-level risk factors (age, orphan status); household risk factors (adults with chronic illnesses and disabilities, greater household size); and household protective factors (including asset-holding). Interactions were systematically tested.

**Results:**

Orphan status was associated with decreased likelihood for birth registration, and paternal orphans and children for whom both parents’ survival status was unknown were less likely to attend school. In the UCT arm, paternal orphans fared better in likelihood of birth registration compared with non-paternal orphans. Effects of study arms on outcomes were not moderated by any other risk or protective factors. High household asset-holding was associated with decreased likelihood of child’s chronic illness and increased birth registration and school attendance, but household assets did not moderate the effects of cash transfers on risk or protective factors.

**Conclusion:**

Orphaned children are at higher risk for poor social protection outcomes even when cared for in family-based settings. UCT and CCT each produced direct effects on children’s social protection which are not moderated by other child- and household-level risk factors, but orphans are less likely to attend school or obtain birth registration. The effects of UCT and CCT are not moderated by asset-holding, but greater household assets predict greater social protection outcomes. Intervention efforts need to focus on ameliorating the additional risk burden carried by orphaned children. These efforts might include caregiver education, and additional incentives based on efforts made specifically for orphaned children.

## Background

Evidence is mounting regarding the beneficial effects of cash transfer programmes in ameliorating children’s health and social risks throughout Latin America, Africa, and Southeast Asia [1]. In Sub-Saharan Africa (SSA), cash transfers have been shown to improve outcomes for children made vulnerable by HIV/AIDS [2, 3]. SSA is disproportionately affected by high prevalence of HIV/AIDS-related maternal mortality [4], and, in recent years, has experienced steep increases in the numbers of orphaned and vulnerable children (OVC) related to HIV/AIDS [5]. Often framed in the context of human rights [6], recent studies of cash transfers in SSA have demonstrated positive impacts on youth HIV risk [2], OVC health outcomes [7], and social outcomes such as school attendance [3, 8] and birth registration [3].

Yet, little is known about whether and how the effects of cash transfers – targeted to households – on improved individual children’s outcomes [9, 10] vary according to underlying children’s risk factors. Some have suggested that children can experience further vulnerability and exclusion even within households, based on gender, disability, or other factors including orphanhood [11]. Disentangling the differential impact of interventions targeted at households is thus an important means of exploring equitability for children at risk of further marginalization.

In terms of protective factors, household asset-holding has been shown to be a buffer against economic shocks which mitigates poor education and health outcomes for children [12]. For vulnerable children and the households which care for them, however, a number of additional risks exist in terms of health vulnerability, household disease burden, and access to material and social supports [13] which asset-holding may or may not offset.

In SSA, the magnitude of the HIV/AIDS crisis has eroded familial networks which traditionally cared for OVC [14] and has posed heightened economic and social risks to households [15]. Among families that support OVC in economically poor communities, some of the most common unmet needs include education, food, medical care and clothing [16] as well as a lack of birth certificates which poses a barrier to accessing school enrollment and health services [17]. Households that have assets tend to have the advantage of having a buffer that mitigates poor outcomes in terms of education and health [9, 18].

Children in asset-poor households are more susceptible to poor health and educational outcomes. These households lack access to medical facilities in the community and resources to pay for medical care [19]. Studies of social assistance in SSA have shown that cash transfers help offset the risk of negative health outcomes for households with the fewest assets [20, 21]. Similarly, children in asset-poor households tend to experience lower educational attainment [22] although this pattern is not universal [23, 24].

For economically poor households in low- and middle-income countries, cash transfer programmes are increasingly used to promote positive health and social outcomes [25]. Many studies have demonstrated positive impacts on reducing a number of social and health vulnerabilities [1, 26, 27]. Conditional cash transfers (CCT), which assign conditions to receiving funds, have been shown to be effective in improving young children’s health and nutritional outcomes [24] and reducing child mortality [28] in Latin America. Unconditional cash transfers (UCT) have been more commonly implemented in sub-Saharan Africa (SSA) [18], although in Zimbabwe, CCTs were shown to be most effective in increasing birth registration and school attendance, with no effect on vaccinations [3].

What remains unexplored in the literature on cash transfers and asset-building is the extent to which individual children’s risk factors moderate the effects of cash transfers on health and social vulnerability. The current study builds on the primary analysis of household data [3] and on analyses of baseline data which showed positive correlations between household asset-holding and OVC social protection [18]. In this study, OVC are defined as children who have lost one or both parents (i.e., single or double orphans), or non-orphaned children who are living in a household that cares for orphans. This study is guided by the following research questions:

1. Which orphan groups - including paternal, maternal, double, and orphans with unknown parents’ survival status - are at greatest risk for health and social vulnerability?
2. To what extent do individual children’s risk factors moderate the impact of UCTs and CCTs on health and social outcomes?
3. To what extent do household assets moderate: (a) the effects of child-level risk factors for children’s health and social outcomes, and (b) the impact of UCTs or CCTs on children’s health and social outcomes?

## Methods

### Sample

A community-randomized controlled trial of UCTs and CCTs was conducted in Manicaland Province, Zimbabwe in 2009 and 2010. Identification of vulnerable households was accomplished by completing a rapid baseline census survey in 10 geographically distinct sites within 3 districts: Nyanga, Makoni and Mutasa [29]. A household is defined as individuals who live within the same homestead and eat from the same pot. Households were identified as ‘vulnerable’ and eligible to participate in the cash transfer trial if they contained children under the age of 18, were not in the wealthiest 20 % of households, and met one or more of the following criteria: (1) were in the poorest quintile of households within each site (bottom 20 %); (2) had one or more orphans; (3) the household head was under age 18; (4) at least one member was chronically ill; or (5) at least one member is disabled. This survey was followed by a community verification exercise in which household reports on eligibility criteria were confirmed or otherwise at community meetings. Only those households identified as eligible in both processes (the BRTI/IC census and the community verification) were treated as eligible for inclusion into the programme. The study was approved by the Medical Research Council of Zimbabwe (MRCZ/A/1518) and the Imperial College Research Ethics Committee (ICREC_9_3_10).

Of the 11,820 households completing a baseline census questionnaire, 10,536 (89 %) reported caring for at least one child and a total of 29,442 children aged 0–17 years were enumerated in the census. Of these households, 4,043 were determined to be eligible and randomized to one of the three trial arms, and 3,818 (94 %) of these were followed up 1.5 years later. Households in the UCT and CCT arms received US$18 plus $4 per child in the household up to a maximum of 3 children. Heads of households received payments from designated locations every 2 months. Households in the CCT arm also were monitored for compliance with several conditions: applying for a birth certificate within 3 months for all children younger than 18 years; for children under 5 (not applicable for the current sample), maintaining up-to-date vaccinations and attending growth monitoring clinics twice a year; for children ages 6–17, school attendance for at least 90 % per month; and, a representative from every household was required to attend two-thirds of local parenting skills classes held by the implementing partner of the project.

For the current study, the sample was narrowed to 2,078 households who were caring for school-aged children ages 6–17 (N = 8,797 children). Of these 8,797 children, 31 were deceased, 396 were lost or were new to the study, and 514 left the household and were not follow-up with at the second wave of data collection, resulting in a sample of 7,857 children. From this sample, 7 % were missing school attendance data, 3 % were missing birth registration status, 8 % were missing chronic illness data, and 8 % were missing chronic disability data. Less than 1 % of data were missing on other covariates, after accounting for cases missing dependent variables. The final sample included 5,331 school-aged children within 1,697 households.

We conducted student’s t-tests to compare the eligible sample (N = 8,797) with the final sample (N = 5,331). The final sample included a higher percentage of participants in the UCT (*p* < .001) group and fewer participants in the control group (*p* < .001), a pattern which suggests greater attrition in control vs. treatment groups over time. There was also a greater percentage of paternal orphans (*p* < .01) and a lower percentage of orphans whose parental survival status was unknown (*p* < .001) in the final sample. The lower attrition rate among paternal orphans may be explained by research which has noted differential outcomes of paternal orphans as mothers tend to be more involved in their child’s wellbeing [30]. Higher attrition for unknown orphan status may be related to less parent involvement. Children tended to be younger in our study sample, compared with the eligible sample (12.07 vs. 11.16, respectively; *p* < .001). The final sample also included fewer children with disability - again likely due to increased attrition from this population (*p* < .001). While these differences suggest differential attrition based on individual and household factors, studies in global contexts have indicated that attrition bias does not dramatically change equation estimates [31, 32].

### Measures

This study included measures of household demographics, child characteristics, and outcome measures. Household demographics included the number of adults and children in the household, the average age of adults in the household, dichotomous measures of adult chronic illness and disability, and socioeconomic status. Socioeconomic status (SES) was measured according to an asset index using a summed score that ranged from 0.01 to 1.00 (higher indicates more assets) and has been used for previous analyses of data from this population [33]. This measure was then used to create asset terciles, representing low, middle, and high asset groups for this population. Child characteristics included child biological sex, age, and orphan status. Orphan status was coded as paternal only (yes/no), maternal only (yes/no), and double orphan (both maternal and paternal; yes/no). Additionally, dummy variables were created to indicate unknown (one or both parents) orphan status, meaning these children may be orphaned or abandoned by parents. Outcome measures included child chronic illness and disability, birth registration status, and school attendance. Chronic illness was defined as being very sick for at least 3 months during the past 12 months, where “very sick” is defined as being too sick to work or do normal activities around the house (coded yes/no). Disability was defined as an impairment that seriously affects ability to perform routine work, school and/or other daily functions, and included such conditions as blindness or visual impairment, deafness, paralysis, physical disability, or mental disturbances; any impairment in these areas resulted in a coding of disability (yes/no). Dichotomous variables birth registration and attending school at least 90 % of days in the past month were each coded yes/no [3].

### Analysis

Independent samples t-tests were conducted to compare pre-post measures within each of the three treatment arms (CCT, UCT, control). Four generalized linear mixed logit models were used to model study outcomes health vulnerability (child chronic illness and disability) and social protection (birth registration and school attendance). Models were estimated as follows, including (1) a time-level sampling model, (2) a time-level link function, and (3) the structural model [34].

1. Sampling model: *E*(*Y*_*ti*_|*u*_*ti*_) = *u*_*ti*_ ; *Var*(*Y*_*ti*_|*u*_*ti*_) = *σ*^2^
2. Link function: *u*_*ti*_ = *η*_*ti*_
3. Structural model:

$$ \begin{array}{l} Time\kern0.50em  level:\kern1em {\eta}_{ti} = {\pi}_{0i} + {\pi}_{1i}tim{e}_{ti} + {\pi}_{2i} Achroni{c}_{ti}+{\pi}_{3i} ADisabilit{y}_{ti}+\\ {}{\pi}_{4i}Aag{e}_{ti} + {\pi}_{5i} NumChildre{n}_{ti}+{\pi}_{6i} Assets{M}_{ti}+{\pi}_{7i} Assets{H}_{ti}+{\pi}_{8i} Cnumbe{r}_{0i}+\\ {}{\pi}_{9i} Cage+{\pi}_{10i} PtOrph+{\pi}_{11i} MOrph+{\pi}_{12i} DbOrph+{\pi}_{13i} UnOrph + \\ {}{\pi}_{14i} TuOrph + {e}_{ti}\end{array} $$

$$ Household\kern0.50em  Level:\kern1em {\pi}_{0i} = {\beta}_{00}+{\beta}_{01}UC{T}_{01}+{\beta}_{01}CC{T}_{01}+{r}_{0i} $$

$$ {\pi}_{1i} = {\beta}_{10}+{r}_{1i} $$

$$ {\pi}_{2i} = {\beta}_{20}+{r}_{2i} $$

$$ {\pi}_{xi} = {\beta}_{x0}+{r}_{xi} $$

Intraclass correlation coefficients (ICC) were calculated for each model to test for within-group variance at the household and child levels, with time (pre/post) nested within children nested within households. Model predictors included time (pre/post), trial arm assignment (UCT and CCT, with control as the reference group), number of children and adults in the household, average adult age, adult chronic illness and disability, SES tercile (low as reference group), child biological sex, child age, and child orphan status (non-orphan status as the reference group). Each of these predictors was systematically tested for interactions with assignment and asset tercile to examine the moderating influence of these variables on health vulnerability and social protection. Study analyses used the xtmelogit command in Stata 12 to fit mixed-effects models for binary outcomes.

## Results

Table 1 includes household-level predictors, child-level predictors, and outcome measures, by assignment (Control, UCT, CCT) and time (pre/post). There was a significant decrease in the average number of adults in households (*p* < .05) and the percentage of adults with chronic illness (UCT *p* < .05; control CCT *p* < .001) in all groups, possibly related to increasing uptake of antiretroviral therapy (ART). There was a significant decrease in adult disability (*p* < .05) in the control and UCT group. Total mean assets increased for each assignment arm (*p* < .001) but, in the control arm, the percentage of households in each asset tercile remained the same. In the UCT group, there was a significant decrease in the percentage of households in the low SES tercile (*p* < .001) while those in the highest tercile significantly increased (*p* < .001). Similarly, in the CCT group, a significant decrease in the percentage of households in the low SES tercile (*p* < .001) was observed while there was a significant increase in the percentage of households in the middle tercile (*p* < .001). Mean child age was approximately 11 years old at the start of the intervention. Over half of the children were not orphans and the frequencies of different forms of orphanhood were consistent between study arms and between time points. The percentage of double “unknown” orphans increased significantly in the control group (*p* < .05) but remained low. Nearly half of the orphaned children were paternal orphans, a quarter were maternal orphans, and a sixth were double orphans, while fewer than 10 % were single unknown orphans and fewer than 1 % were double unknown orphans. No significant changes over time were observed in either child disability or chronic illness across assignment groups. Both birth registration and school attendance increased in all groups (*p* < .01).

**Table 1:** Household-level and Child-level Pre-post Measures with Levels of Significance

	Control (*N*=1,478)		UCT (*N*=2,035)		CCT (*N*=1,818)	
Covariate	Pre	Post	Pre	Post	Pre	Post
Household Characteristics						
Average adult age	39.80	41.19**	41.30	42.73**	40.2	41.65***
Number of adults	2.95	2.83*	2.87	2.76*	2.90	2.79*
Number of children	3.56	3.64	3.57	3.69	3.78	3.88
Adult chronic illness	.47	.36***	.50	.45*	.54	.39***
Adult disability	.14	.12*	.13	.10*	.14	.13
SES tercile low	.38	.38	.46	.40***	.46	.40***
SES tercile mid	.24	.23	.24	.23	.20	.25***
SES tercile high	.38	.40	.30	.37***	.34	.35
Total Assets	.19	.20***	.18	.20***	.18	.19***
Child Characteristics^a^						
% Female	.50	-	.49	-	.49	-
Age	11.07	-	11.11	-	11.28	-
Not orphan	.56	.53	.56	.56	.56	.57
Paternal orphan	.47	.49	.47	.48	.47	.48
Maternal orphan	.24	.26	.24	.26	.24	.25
Double orphan	.16	.17	.16	.18	.16	.17
Unknown, 1 parent	.08	.09	.08	.09	.08	.07
Unknown, 2 parent	.001	.007*	.003	.002	.003	.006
Outcome Variables						
Child with disability	.02	.02	.01	.01	.02	.02
Chronic illness	.05	.05	.06	.06	.06	.06
Birth registration****	.76	.82***	.77	.86***	.74	.89***
School attendance****	.75	.80**	.75	.90***	.76	.93***

*N* children = 5,331

*N* households = 1,697

*p < .05 for pre-post, two-tailed **p < .01 for pre-post, two-tailed ***p < .001 for pre-post, two-tailed, ****p < .001, ANOVA difference among values at time = post

^a^Orphan categories listed in pre-post measures are not mutually exclusive

### Health Vulnerability

Four generalized mixed linear models are presented in Table 2, one for each outcome variable. The first two models predicted *health vulnerability* as measured by child disability and child chronic illness. Orphan status was not significantly associated with child disability. 1Middle SES tercile was associated with increased odds of disability (*p* < .05), while female children had lower odds of disability (*p* < .05). The main effect of the unconditional cash transfer (UCT) treatment arm significantly predicted lower odds of child disability (*p* < .05) though this should be interpreted with caution as the interaction of UCT and time was not significant. No interactions between assignment, assets, and other covariates showed statistically significant results.

**Table 2:** Generalized linear mixed models predicting health vulnerability and social protection

	*Health vulnerability*				*Social protection*			
Variable	Child with disability		Chronic illness		Birth registration		School attendance	
	OR	CI	OR	CI	OR	CI	OR	CI
*Fixed Effects*								
*Assignment*								
Time	1.14	.58–2.29	1.19	.80–1.77	1.41*	1.05–1.88	1.53***	1.26–1.86
(Control)								
UCT	.37*	.15-.94	1.33	.88–2.01	1.07	0.71–1.61	1.03	.85–1.26
CCT	1.17	.51–2.7	1.11	.72–1.71	.81	0.55–1.2	1.24*	1.00–1.52
UCT x Time	1.27	.47–3.41	.85	.51–1.40	1.50*	1.03–2.2	2.50***	1.90–3.29
CCT x Time	1.06	.43–2.58	1.20	.72–2.01	4.34***	2.88–6.55	3.30***	2.46–4.43
*Household Characteristics*								
Adult chronic illness	1.21	.85–1.72	1.98***	1.68–2.33	.96	0.82–1.13	.73***	.67-.81
Adult with disability	1.00	.51–1.96	1.12	.80–1.55	.67**	0.49–0.9	.80*	.67-.95
Age of adult	.99	.96–1.01	.98**	.97-.99	1.00	0.99–1.01	1.00	1.00–1.01
Number of adults in HH	1.05	.87–1.28	.94	.86–1.04	1.10*	1–1.2	1.05*	1.00–1.11
(SES tercile low)								
SES tercile middle	2.04*	1.12–3.72	.91	.67–1.23	1.70***	1.29–2.24	1.34***	1.14–1.58
SES tercile high	.57	.30–1.08	.70*	.52-.94	2.70***	2.04–3.57	1.75***	1.50–2.05
Number of children in HH	.92	.78–1.09	.82***	.88-.96	.90**	0.83–0.97	.94**	.90-.98
*Child Characteristics*								
Child sex (1 = female)	.56*	.32-.99	.94	.72–1.21	1.26	0.98–1.61	1.04	.92–1.17
Child age	1.05	.95–1.15	.92***	.88-.96	1.45***	1.38–1.52	.95***	.93-.97
Orphan status								
Non-orphan (ref. group)	-	-	-	-	-	-	-	-
Paternal orphan	.58	.30–1.10	.90	.66–1.22	.63*	0.45–0.9	.76**	.66-.89
Maternal orphan	.59	.22–1.58	1.20	.76–1.92	.24***	0.16–0.35	.83	.65–1.06
Double orphan	.68	.31–1.49	1.37	.95–1.98	.25***	0.18–0.36	1.17	.86–1.58
Unknown, 1 parent	1.51	.62–3.65	1.23	.80–1.90	.33***	0.23–0.48	.94	.75–1.18
Unknown, 2 parents	-	-	.30	.02–4.23	.03***	0.01–0.1	.39*	.17-.92
Constant	.00	.00-.00	.06	.02-.18	23*	0.1–0.54	5.18***	3.20–8.40
*Interactions*								
CT x Pat. orphan	-	-	-	-	1.71*	1.01–2.92	-	-
*Random Effects*								
Household Intercept	1.92	1.34–2.76	.96	.70–1.32	2.33	2.32	2.07–2.60	1.05–1.27
Child Intercept	3.14	2.50–3.95	2.05	1.74–2.40	2.46	2.46	2.22–2.72	.00
*Household-level ICC*	.58		. 24		.55		.26	

*N* children = 5,331

*N* households = 1,697

*p < .05 **p < .01 ***p < .001

In the second model (see Table 2), neither orphan status nor treatment arm predicted child chronic illness. Children from high asset households were also less likely to suffer from chronic illness (*p* < .05). Child age (*p* < .001) and number of children in the household (*p* < .001) each predicted lower likelihood of chronic illness. Adult chronic illness increased the odds that their children would suffer from chronic illness themselves (*p* < .001). No interactions between assignment, assets, and other covariates showed statistically significant results.

### Social Protection

The third and fourth models predicted *social protection* as measured by birth registration and school attendance (see Table 2). Maternal orphans (OR = 0.24; CI = 0.16, 0.35; *p* < .001), paternal orphans (OR = 0.63; CI = 0.45, 0.9; *p* < .05), and double orphans (OR = 0.25; CI = 0.18, 0.36; *p* < .001) were less likely to have obtained birth registration, compared to non-orphans. Additionally, children with unknown status of one parent (OR = 0.33; CI = 0.23, 0.48; *p* < .001) or both parents (OR = 0.03; CI = 0.01, 0.10; *p* < .001) were much less likely to be registered (p < .001). Compared to the lowest assets tercile, middle tercile children (OR = 1.70; CI = 1.29, 2.24; *p* < .001) and high tercile children (OR = 2.70; CI = 2.04, 3.57; *p* < .001) were both more likely to be registered.

In contrast to the first two models, as reported in the primary analysis of trial results, [5] the interaction of the CCT treatment arm and time (*p* < .001) significantly predicted birth registration status, with the children of CCT households being significantly more likely to obtain birth registration, controlling for other factors (OR = 4.34; CI = 2.88–6.55; *p* < .001). Children in the UCT group were also more likely to obtain birth registration, controlling for other factors (OR = 1.50; CI = 1.03-2.20; *p* < .05); this finding stands in contrast to previous research with this sample [5] but likely reflects this study’s focus on school-aged children as well as an analytical focus on all children in households as units of analysis. Greater number of children in the household (*p* < .01) predicted a lowered likelihood for registration, while child’s age (*p* < .001), was associated with increased likelihood for registration.

**Fig. 1:**
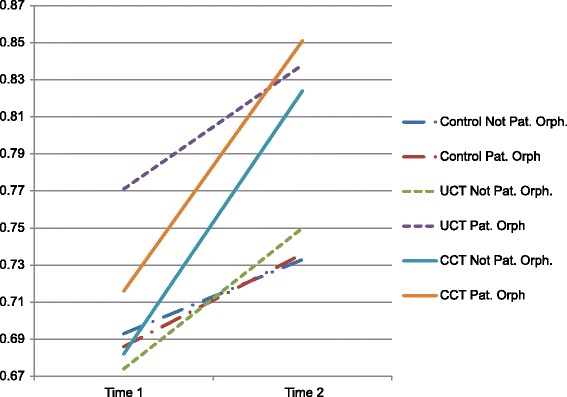
Predicted percent birth registration, paternal orphan status by assignment

Although interactions between children’s risk factors and study arms were systematically tested, the interaction between UCT and paternal orphan status was the only significant interaction which emerged in any model (OR = 1.71; CI = 1.01, 2.92; *p* < .05). Fig. 1 presents the results of the interaction of paternal orphan status by assignment across time points. For both the UCT and CCT arms, but not in the control arm, predicted values of birth registration were higher among paternal orphans in comparison to others (non-orphans and other orphan types) at both time points, though this relationship was only significant for the UCT group. Birth registration increases over time in the UCT and CCT arms for both paternal orphans and others, but the relative advantage of paternal orphans remains about the same. In the control arm, paternal orphans have a slight disadvantage compared with others which disappears at follow-up, although both groups show less improvement compared with UCT and CCT arms.

In the fourth model, older children (*p* < .001) and paternal orphans (*p* < .01) were less likely to attend school. Compared to children in the lowest SES tercile, children in middle SES households (OR = 1.34; CI = 1.14–1.58; *p* < .001) and those in high SES households (OR = 1.75; CI = 1.50–2.05; *p* < .001) were more likely to attend school. The interaction between time and assignment was significant for both the UCT (OR = 2.50; CI = 1.90, 3.29; *p* < .001) and CCT (OR = 3.30; CI = 2.46, 4.43; *p* < .001) treatment arms. Among household characteristics, adult chronic illness (*p* < .001), adult disability (*p* < .05), and number of children (*p* < .05) were risk factors for lowered school attendance, while number of adults in the home (*p* < .05) predicted greater school attendance. No interactions between assignment, assets, and other covariates showed statistically significant results.

## Discussion

In the context of HIV/AIDS, previous research on orphaned and vulnerable children suggests that losing one or both parents significantly increases vulnerability for children [35]. Our study showed that orphan status significantly predicted heightened social vulnerability but not health vulnerability. Maternal and paternal orphans are at risk for not obtaining birth registration, while UCT buffers this risk for paternal orphans. These differences may imply that the registration process is a gendered activity, whereby women take on a more central role in the registering their child – a role that is not replaced by receipt of cash assistance alone. Mothers of paternal orphans may use the incentives of the cash transfer to invest in the human capital of their children at greater rates than children in other household circumstances. Importantly, these findings reinforce the need to focus on the potential of marginalization within households, based on gender or orphanhood [11], to maximize children’s social protection.

Paternal orphans (but not maternal orphans), however, were at greater risk of not attending school. This finding stands somewhat in contrast with an earlier study in this region which found that maternal orphans were most likely to be out of school [36]. This difference may be attributable to the significant economic changes happening in Zimbabwe during the interim, or possibly the effects of OVC programming in the area. Nevertheless, the increased risk for paternal orphans possibly suggests that children in households without a male parent may be pressured to take on domestic responsibilities or enter the labor force in order to support the family.

Children with two parents whose survival status is unknown were significantly less likely to obtain birth registration or attend school, patterns which suggest that these children are at heightened risk. Specifically, children with one parent of unknown status were less likely than paternal orphans to have a birth registration, while children with both parents of unknown status were much less likely to be registered in comparison to double orphans. Children of unknown orphan status in the first years of their lives are not only at risk for having lost one or more of their parents; the unknown status of their parents prevents them from obtaining the birth registration that will be required throughout their lives to access important social services.

Further research is needed to explore whether unknown status is a definitional problem [37] or if this sub-group represents a new population of concern who are possibly abandoned by parents [38]. Some of these children’s caregivers may be distant relatives who did not have much contact with them, or their parents, prior to children’s residing in the household. Alternatively, some children may be living with grandparents where the parents may have died, or moved elsewhere; and some children may be living with single parents whose partners left, or were never engaged, and thus have little support in raising children.

One of the main questions this study answers is the extent to which cash transfers influence OVC well-being directly, or whether households’ accumulation of assets serves as a greater buffer against health and social vulnerability for school-aged children [19]. The answer to this question is that cash transfers and asset-holding each have direct effects on OVC birth registration and school attendance, while assets alone have a weak but positive effect on children’s chronic illness and no effect on chronic disability. Thus, while both assignment and assets increase the odds of OVC social protection individually, this increase does not change depending on the amount of assets held by the household.

As in prior research, higher asset-holding [17] predicts more positive health outcomes, although weakly and only for chronic illness. Contrary to other studies [20, 21], however, cash transfers seem to have no significant effect on health vulnerability. It is possible that those households with the most assets, while still poor relative to the wider community above the lowest wealth quintile, are able to use assets to access health care in treating OVC chronic illness. Alternatively, those households with greater assets may also experience better living conditions more generally which contribute to better health outcomes. Given the relatively short amount of time between baseline and follow-up, it may be that not enough time had elapsed to detect the potential impact of cash transfers or to allow for changes in long-term health outcomes.

Both UCT and CCT significantly improved birth registration and school attendance for children ages 6–17 years old. Neither intervention arm moderated household or child-level risk factors, except that paternal orphans fared better than non-orphans in the UCT arm. These findings contribute further evidence to the effectiveness of cash transfers in promoting social protection for vulnerable populations [3, 19] even when controlling for heightened risk. Yet, birth registration and school attendance increased for all groups, including the control group. The baseline study occurred immediately after a period of economic collapse in Zimbabwe. The background increases in use of services at follow-up likely reflect the recovery of the economy after the introduction of the multi-currency system early in 2009.

Households with more asset holding were associated with less social vulnerability. In our study, the evidence suggests that the additional income provided by cash transfers provides a substantial boost to improved OVC outcomes, especially social protection. Alternatively, the additional support to households, which accompanies the intervention, may also provide a protective influence on children’s outcomes.

Significantly, household disease burden also plays an important role [13]; greater number of chronically-ill adults predicts greater children’s chronic illness even controlling for cash transfers and assets. One explanation may be that chronic illness is shared between adults and children, given the larger context of a major HIV epidemic with limited prevention of mother-to-child transmission (PMTCT) services. Another explanation may be that chronically-ill adults lack the ability to access needed medical care for children in the household, resulting in higher incidences of disease and chronic illness. This explanation is somewhat less plausible, given that the Government of Zimbabwe and UNICEF sponsor outreach vaccination campaigns with reasonable coverage levels, and healthcare costs at child clinics tend to be free or cheap, and accessible by foot.

The finding that older children were more likely to receive birth registration is somewhat surprising, given the expressed commitment of the Zimbabwean government in promoting birth certification for children as a human right [39]. This dynamic may reflect a deterioration of civil services in the wake of the financial crises in Zimbabwe occurring immediately prior to this study. An alternative explanation is that UCT and CCT are an effective means of reducing barriers to older children’s receiving birth registration, particularly in rural areas where rates of registration tend to be lower [40].

Clustering of children’s outcomes within households accounted for more variance in social protection than for health vulnerability. This pattern may imply that intervening at the household level may be more tractable for improving social outcomes. Improving health outcomes may require child-specific targeting efforts.

### Limitations

A limitation of this study is the short period of time between baseline and follow up, such that the longer-term trajectories related to treatment arms are unclear. The study also was conducted shortly after a period of severe economic crisis in Zimbabwe, a context which may affect the generalizability of the findings. The reason behind unknown orphan statuses is unclear; given the poorer outcomes experienced by OVC with unknown status, further exploration of this subgroup is warranted.

## Conclusion

Orphaned children are at higher risk for poor social protection outcomes even when cared for in family-based settings. Orphans remain at higher risk for not obtaining birth registration despite the general improvement brought about by cash transfers, particularly those for whom parent status remains unknown. However, the benefits of UCT were greater for paternal orphans. UCT and CCT each produced direct effects on children’s social protection which are not moderated by other child- and household-level risk factors. The effects of UCT and CCT are not moderated by asset-holding, but greater household assets predict greater social protection outcomes. Intervention efforts need to focus on ameliorating the additional risk burden carried by orphaned children. These efforts might include caregiver education, and additional incentives based on efforts made specifically for orphaned children.

## Footnotes

1 Double-unknown parent status was not estimated in the disability model as there was no variation in the dependent variable (i.e., no double-unknown orphan status children had a disability).
